# Endothelial Differentiation of Human Stem Cells Seeded onto Electrospun Polyhydroxybutyrate/Polyhydroxybutyrate-Co-Hydroxyvalerate Fiber Mesh

**DOI:** 10.1371/journal.pone.0035422

**Published:** 2012-04-16

**Authors:** Alessandra Zonari, Silviene Novikoff, Naira R. P. Electo, Natália M. Breyner, Dawidson A. Gomes, Albino Martins, Nuno M. Neves, Rui L. Reis, Alfredo M. Goes

**Affiliations:** 1 Department of Biochemistry and Immunology, Federal University of Minas Gerais, Belo Horizonte, Minas Gerais, Brazil; 2 3B's Research Group - Biomaterials, Biodegradables and Biomimetics, University of Minho, Guimarães, Portugal; 3 ICVS/3B's - PT Government Associate Laboratory, University of Minho, Braga/Guimarães, Portugal; Université de Technologie de Compiègne, France

## Abstract

Tissue engineering is based on the association of cultured cells with structural matrices and the incorporation of signaling molecules for inducing tissue regeneration. Despite its enormous potential, tissue engineering faces a major challenge concerning the maintenance of cell viability after the implantation of the constructs. The lack of a functional vasculature within the implant compromises the delivery of nutrients to and removal of metabolites from the cells, which can lead to implant failure. In this sense, our investigation aims to develop a new strategy for enhancing vascularization in tissue engineering constructs. This study's aim was to establish a culture of human adipose tissue-derived stem cells (hASCs) to evaluate the biocompatibility of electrospun fiber mesh made of polyhydroxybutyrate (PHB) and its copolymer poly-3-hydroxybutyrate-co-3-hydroxyvalerate (PHB-HV) and to promote the differentiation of hASCs into the endothelial lineage. Fiber mesh was produced by blending 30% PHB with 70% PHB-HV and its physical characterization was conducted using scanning electron microscopy analysis (SEM). Using electrospinning, fiber mesh was obtained with diameters ranging 300 nm to 1.3 µm. To assess the biological performance, hASCs were extracted, cultured, characterized by flow cytometry, expanded and seeded onto electrospun PHB/PHB-HV fiber mesh. Various aspects of the cells were analyzed *in vitro* using SEM, MTT assay and Calcein-AM staining. The *in vitro* evaluation demonstrated good adhesion and a normal morphology of the hASCs. After 7, 14 and 21 days of seeding hASCs onto electrospun PHB/PHB-HV fiber mesh, the cells remained viable and proliferative. Moreover, when cultured with endothelial differentiation medium (i.e., medium containing VEGF and bFGF), the hASCs expressed endothelial markers such as VE-Cadherin and the vWF factor. Therefore, the electrospun PHB/PHB-HV fiber mesh appears to be a suitable material that can be used in combination with endothelial-differentiated cells to improve vascularization in engineered bone tissues.

## Introduction

Tissue engineering is ‘an interdisciplinary field that applies the principles of engineering and life sciences toward the development of biological substitutes that restore, maintain, or improve tissue function or a whole organ’ [1] . One approach utilized in this area is based on the fabrication of scaffolds that can be employed as cell support devices upon which cells are seeded *in vitro* and able to lay down a matrix to produce the foundations of a tissue for transplantation. Growth factors can be introduced to stimulate cell proliferation and differentiation [2], [3].

The need for bone repair in many patients suffering from large bone resections or significant trauma has led to the development of bone grafts using tissue-engineering approaches. To become widely used in clinical practice, bone tissue-engineering products must overcome a series of challenges, the completely supply of nutrients and metabolites diffusion being one of the most important. [4]–[6]. Indeed, the vascularization of cell-seeded implants plays an important role in cell survival, as these cells require access to substrate molecules (oxygen, glucose and amino acids) and clearance of products of metabolism (CO_2_, lactate and urea) [7], [8]. Additionally, due to the limitations of oxygen diffusion, most cells cannot survive at distances greater than or equal to 150 µm from a capillary [4] and blood vessels also have important metabolic and rheological functions that are organ-specific and important for the regeneration of tissue [9].

Therefore, as in the development of native bone, vascularization following implantation is of critical importance for the survival, integration and functionality of engineered bone tissue [10]. Many approaches have been proposed to increase vascularization in bone tissue engineering, including modulation of scaffold architecture, inclusion of angiogenic growth factors, co-culture systems and pre-vascularization *in vitro* [11]–[17].

In blood vessels, endothelial cells are attached as a monolayer to a basement membrane composed of protein fibers at the nanoscale, such as type IV collagen and laminin fibers. This natural extracellular matrix (ECM) is critical for the support of the vascular endothelium by maintaining the organization of vascular endothelial cells into blood vessels. Furthermore, the endothelial cells proliferation, migration, morphogenesis, survival and blood vessel stabilization are dependent on their adhesion to the ECM [18].

In the biomaterials field, the electrospinning processing technique allows to produce polymer fibers with diameters in the range of nanometers to micrometers that are physically comparable to the collagen fibers found in the natural ECM and [19], [20] has been extensively employed in tissue engineering strategies [21]–[26].

Polymeric biomaterials have widely been utilized in biomaterials research for tissue engineering applications, mainly because of the great variety of natural and synthetic biodegradable polymers. [27].

Polyhydroxyalkanoates (PHAs) are naturally polyesters of hydroxyalkanoic acids that are synthesized by a wide range of bacteria, generally under unbalanced growth conditions, to act as carbon and energy reserve materials [28]. PHAs are promising materials for various applications, including tissue engineering, because they have useful mechanical properties and are biodegradable and biocompatible [29]. In fact, polyhydroxybutyrate (PHB) and poly(3-hydroxybutyrate-co-3-hydroxyvalerate) (PHB-HV), which are members of PHAs family, degrade *in vivo* into D-3-hydroxybutyrate, a normal constituent of human blood [30]–[32].

Although PHB is inherently biocompatible and biodegradable, the use of PHB is significantly limited in biomedical applications by several of its characteristics, including rigidity, brittleness and low mechanical properties [33], [34]. PHB-HV is less crystalline and more flexible than PHB [35], [36]. The combination of these two polymers could provide a suitable candidate material blend for bioactive and biodegradable composite implants that would guide tissue growth and be replaced by newly formed tissue [37].

The aim of the current study was to create an endothelial network that could improve the vascularization of engineered bone tissue. To this end, we established a culture of human adipose tissue-derived stem cells (hASCs) to evaluate the biocompatibility of electrospun fiber mesh made of PHB and PHB-HV and to promote the differentiation of hASCs into endothelial cells.

## Results

### Morphology of electrospun PHB/PHB-HV fiber mesh

**Figure 1:**
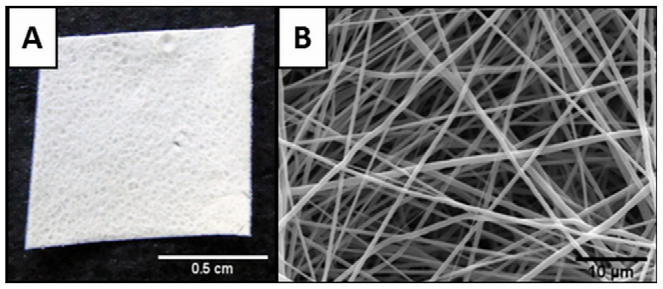
Structure of the electrospun PHB/PHB-HV fiber mesh. (A) Digital photograph of the electrospun PHB/PHB-HV fiber mesh surface; (B) SEM micrograph illustrating the disposition of the fibers.

The PHB/PHB-HV fiber mesh was produced by electrospinning from a blend of 30% PHB and 70% PHB-HV. Figure 1A depicts the top surface of the electrospun fiber mesh. The SEM image confirmed the possibility of obtaining a mesh-like structure with 100 µm thickness and composed of randomly distributed fibers with diameters ranging from 300 nm to 1.3 µm with mean of 770 nm±0,25 (Figure 1B). This structure is similar to that of a natural ECM.

### Isolation and characterization of human adipose tissue-derived stem cells

**Figure 2:**
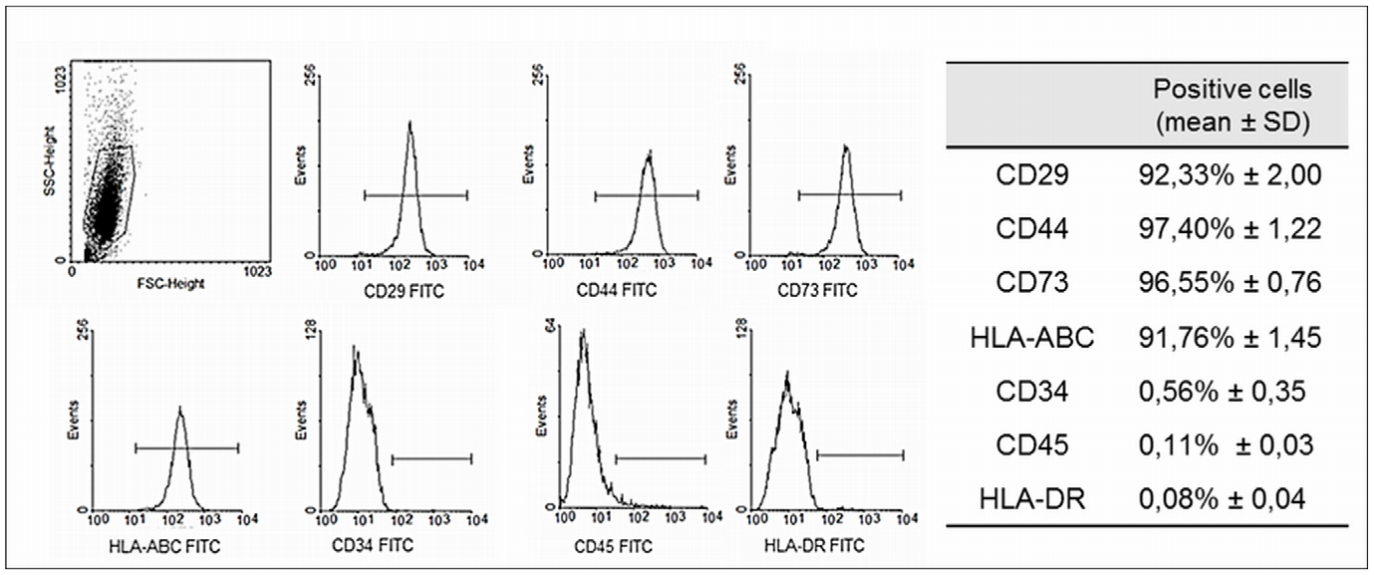
Flow cytometry analysis of hASCs. The expression pattern of specific antigens on the surface of the hASCs is depicted with representative histograms and the expression of each marker. The cell population expressed CD29, CD44, CD73 and HLA-ABC, and did not express CD34, CD45 and HLA-DR.

Stem cells from the stromal fractions of adipose tissue were isolated by enzymatic dissociation following centrifugation. The isolated cells displayed a fibroblastic morphology and were adherent to plastic. Also, the cells were able to self-renew and form colonies (data not shown). Isolated stem cells from human adipose tissue were expanded and their specific surface antigens were characterized by flow cytometry, which was essential for ensuring the purity of the cell population. Flow cytometry analysis indicated that the isolated cell population expressed CD29 (92,33%±2,00), CD44 (97,40%±1,22), CD73 (96,55%±0,76) and HLA-ABC (91,76%±1,45) and was completely isolated from lymphocytes and hematopoietic stem cells, as the cells did not express CD34 (0,56%±0,35), CD45 (0,11%±0,03) and HLA-DR (0,08%±0,04) (Figure 2). This isolated cell population was designed human adipose tissue-derived stem cells (hASCs).

### Cell adhesion and morphology by SEM

**Figure 3:**
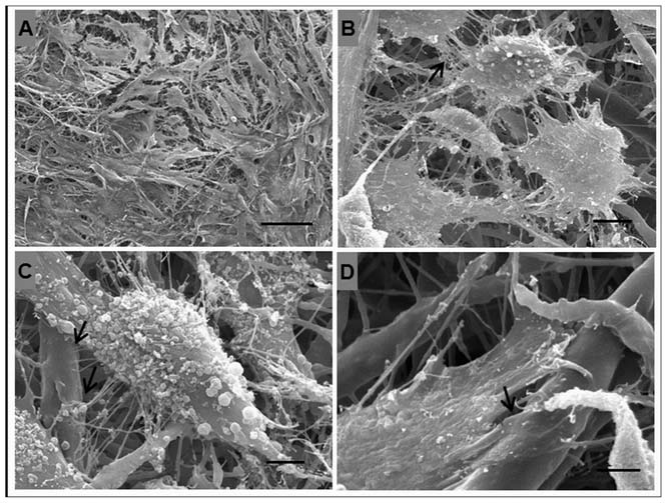
SEM micrographs of the morphology of hASCs cultured for 7 days on the electrospun PHB/PHB-HV fiber mesh. (A) Panoramic view depicting high cell density and spindle-shape morphology, scale bar: 100 µm; (B) arrow indicate the interaction between two and more cells, scale bar: 10 µm; (C–D) arrows indicate the interaction of hASCs with the fibers, scale bar: 5 µm.

Human adipose tissue-derived stem cells (hASCs) were seeded onto the electrospun fiber mesh and cultured for seven days. The morphology of the cells was examined by SEM, which indicated that the cells adhered to and interacted with the fibrous structure and spread over the surface. The cells exhibited the typical spindle-shape morphology and cell-to-cell interactions were also observed (Figure 3).

### Cell proliferation and viability

**Figure 4:**
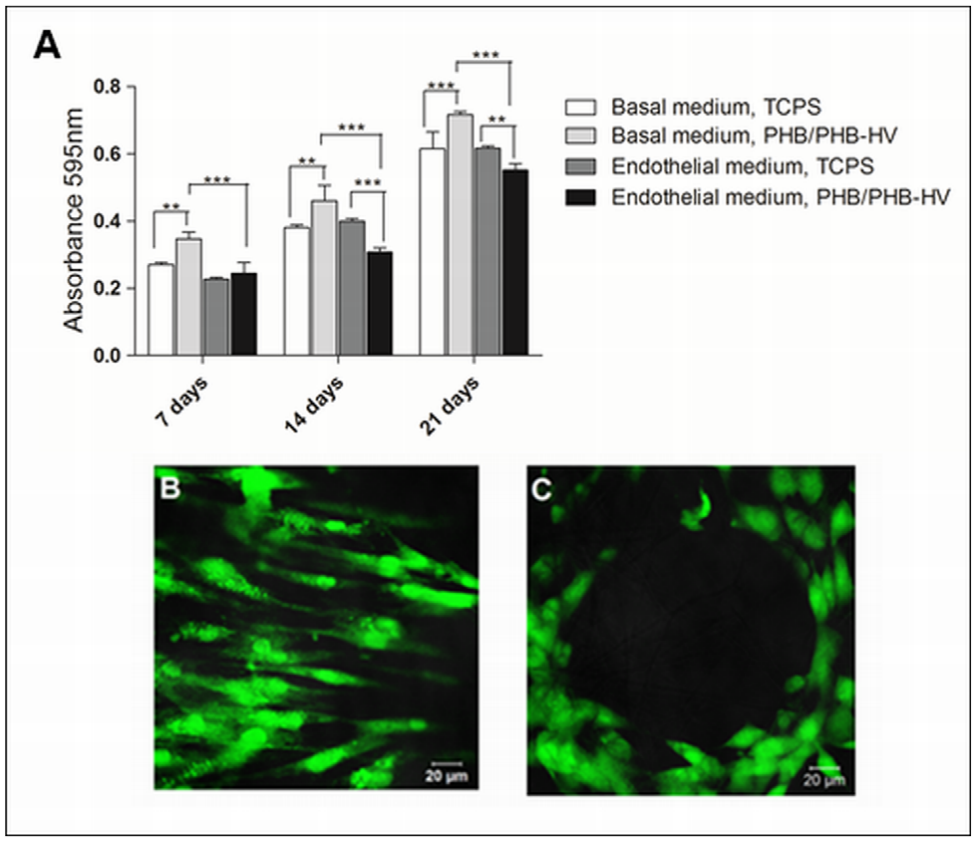
Proliferation and viability of hASCs cultured on TCPS and electrospun PHB/PHB-HV fiber mesh. (A) MTT proliferation assays performed 7, 14 and 21 days after the cell seeding and cultured in two specific medium: the basal medium and the endothelial differentiation medium. The results are expressed as the means ± SD, (*) indicating a significant difference with p<0.05, (**) p<0,01, (***) p<0,001; (B) cell viability after 21 days of cell culture with the basal medium and (C) the endothelial differentiation medium on the electrospun PHB/PHB-HV fiber mesh, as analyzed by Calcein-AM staining.

A metabolic activity-based assay (MTT) was performed at various time points to determine the cells viability and proliferation when cultured in two types of culture medium: a basal medium and an endothelial differentiation medium. MTT results are directly proportional to the number of living cells. The results indicated that hASCs proliferated in both types of medium since the absorbance value enhanced from 7 to 21 days (Figure 4A). When cultured in basal medium, hASC exhibit enhanced proliferation when seeded on electrospun PHB/PHB-HV fiber mesh comparing to culture plates. During the endothelial differentiation, hASCs cultured on electrospun PHB/PHB-HV fiber mesh proliferate less comparing to hASCs cultured on electrospun PHB/PHB-HV fiber mesh with basal medium and hASCs cultured without fiber mesh with endothelial differentiation medium. When the endothelial differentiation was performed in cells cultured without the fiber mesh, no difference on the proliferation comparing with the basal medium was detected (Figure 4A) Moreover, cell viability assay employing Calcein-AM staining was performed to ensure the viability and assess the morphology and distribution of hASCs after 21 days (Figure 4B and C). These results showed that the cells cultured on electrospun PHB/PHB-HV fiber mesh with basal medium are well distributed and the cells cultured on electrospun PHB/PHB-HV fiber mesh with endothelial differentiation medium rearrange themselves forming circle-like structures that are characteristic of endothelial cells organization, mimicking the tubular organization of blood vessels.

### Endothelial differentiation

**Figure 5:**
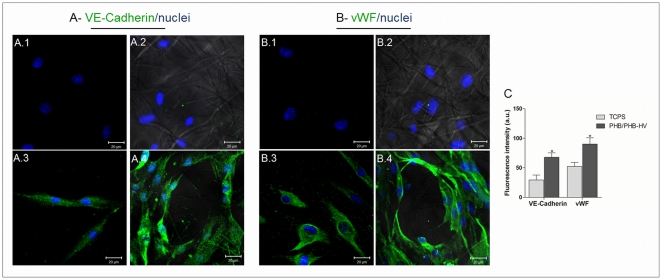
Protein expression of hASCs during endothelial differentiation. Confocal images of the expression of the VE-Cadherin (A) and the vWF factor (B) after 21 days. A.1 and B.1 - hASCs cultured with the basal medium on the TCPS coverslips, A.2 and B.2 - hASCs cultured with the endothelial differentiation medium on the TCPS coverslips, A.3 and B.3 - hASCs cultured with the basal medium on the electrospun PHB/PHB-HV fiber mesh, A.4 and B.4 - hASCs cultured with the endothelial differentiation medium on the electrospun PHB/PHB-HV fiber mesh. Scale bar 20 µm. The images, A.2, A.4, B.2 and B.4 represent the overlay of bright-field and confocal images for visualization of the fiber mesh. (C) Fluorescence intensity of the expression of VE-Cadherin and the vWF factor in cells differentiated on TCPS coverslip and electrospun PHB/PHB-HV fiber mesh. The results are expressed as the means ± SD, (*) significant difference for p<0,05. (a.u): arbitrary units.

**Figure 6:**
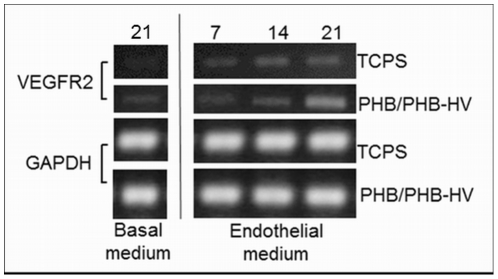
RT-PCR analysis of VEGFR2 mRNA expression during endothelial differentiation on the electrospun PHB/PHB-HV fiber mesh and TCPS. Total RNA was extracted from cells cultured on basal medium and endothelial differentiation medium for analysis of VEGFR2 mRNA expression. The cells were cultivated up to 21 days.

To analyze the cells differentiation towards the endothelial phenotype, an immunofluorescence assay was performed to examine the expression of endothelial markers. The hASCs cultured with basal medium indicated no specific staining for VE-Cadherin and vWF when cultured on TCPS coverslips (Figure 5A) and electrospun PHB/PHB-HV fiber mesh (Figure 5D). After 21 days of culture in endothelial differentiation medium, hASCs expressed VE-Cadherin and vWF when cultured on TCPS coverslips (Figure 5B and C) and electrospun PHB/PHB-HV fiber mesh (Figure 5E and F). When the endothelial induction was performed on hASCs cultivated on electrospun PHB/PHB-HV fiber mesh, the cells exhibited tube-like structures that were not present in hASCs differentiated in TCPS coverslips (Figure 5E and F). Additionally, the cells that were differentiated on electrospun PHB/PHB-HV fiber mesh presented enhanced fluorescence intensity for both markers comparing to cells differentiated on TCPS coverslip (Figure 5H). For more evidence of endothelial differentiation, the mRNA expression over time of selected endothelial marker on hASCs cultured on TCPS and on the electrospun PHB/PHB-HV fiber mesh was analyzed by RT-PCR. The results indicated that the mRNA expression of VEGFR2 was detected in the cells cultured for 21 days in basal medium, but was upregulated after 21 days in endothelial differentiation medium, more evidenced in the cells cultured on electrospun PHB-HV fiber mesh than in TCPS(Figure 6). These results indicate that the cells, cultured on electrospun PHB-HV fiber mesh successfully differentiated into the endothelial phenotype.

## Discussion

Regenerative medicine is based on the possibility of repairing tissues damaged by disease, trauma or senescence. In tissue engineering approaches, these repairs may be achieved by combining the materials field with the life science to create a construct that can temporarily replace mechanical function, guide tissue growth and degrade at rates appropriate to tissue regeneration [1]. Furthermore, for metabolically active tissues, such as the bone tissue, the vascularization of the construct to be implanted is crucial for cell survival and the formation of new tissue [6], [38]. Bone growth greatly depends on the vascular network to supply nutrients, remove metabolites and allow osseous formation and repair. In large bone grafts, without the proper blood supply, seeded osteoblasts in the middle of porous scaffolds are subject to necrosis due to ineffective transportation of oxygen, nutrients and metabolites and only a shell of bone may be formed at the surface of the scaffold. In this way, the rate and range of vascular growth seem to determine the efficiency of new bone formation. Numerous strategies for vascularization of engineered bone tissue have been reported, but most involve sophisticated scaffolds, co-cultures systems and local delivery of angiogenic factors [11]–[17]. Thus, the ability to enhance the vascularization of bone grafts in a clinically relevant manner could vastly expand the utility of engineered bone tissue grafts.

In this study, we developed a fiber mesh made by blending PHB and PHB-HV using the electrospinning technique. These polymers are members of the PHA family and are useful for tissue engineering, due to their biocompatibility and biodegradability. Various scaffolds made by PHB or PHB-HV have demonstrated high levels of biocompatibility and cell attachment with various cell types [39]–[42]. Electrospinning has been proposed as a promising technique to produce fiber mesh with fiber diameters ranging from a few micrometers to the nanometer range [22]. Sombatmankhong and colleagues [37] successfully fabricated and characterized PHB/PHB-HV fibers in various compositions. In contrast to our fiber mesh, that exhibits fibers with diameters ranging from 300 nm to 1.3 µm, the structures obtained by Sombatmankhong and colleagues [37] exhibited fibers with diameters ranging from 1.1 µm to 4.0 µm. The natural extracellular matrix is composed of randomly oriented collagens of nanometer-scale diameters similar to our structure, which favors cell adhesion, proliferation and differentiation. It was also reported the production of electrospun fiber meshes by blending PHB with other biomaterials, such as chitosan [43] and polyvinyl alcohol (PVA) [44]. Both fibers showed to be nontoxic and enable for cell attachment, growth and proliferation.

To assess the biocompatibility of electrospun PHB/PHB-HV fiber mesh, isolated human adipose tissue cells were expanded and characterized by flow cytometry. The isolated cell population exhibited a specific surface antigen expression pattern characteristic of mesenchymal stem cells, which indicated that these cells are human adipose-derived stem cells (hASCs) [45]–[47]. The SEM images indicated that the hASCs attached to the electrospun PHB/PHB-HV fiber mesh and spread extensively. The hASCs were able to interact with the PHB/PHB-HV fibers and with neighboring cells. The hASCs attached at multiple focal points at the surface of the fibers, demonstrating that the cells adapted to this new environment.

The MTT assay is employed to evaluate the cytotoxicity of various tissue engineering materials, and the absorbance value can provide an indication of the cells growth and proliferation. When cultured in basal medium, the hASCs proliferated more on the electrospun PHB/PHB-HV fiber mesh over an increased culture time comparing to hASCs cultured on culture plates. This confirmed that the fiber mesh promotes cell proliferation without a cytotoxic effect and is a better environment for the cells. The viability of the cells was confirmed by the Calcein-AM assay. After 21 days, the cells seeded onto the electrospun PHB/PHB-HV fiber mesh were live cells exhibiting intact membranes. Our results were in line with those of another study that seeded hASCs onto electrospun PLA nanofiber [48]. Furthermore, prior studies have demonstrated that hASCs can grow and proliferate with various types of biomaterials [49]–[52]. Thus, hASCs can be considered a good cell type for a tissue engineering approach, as they are derived from an abundant and available source and can be harvested with minimal morbidity. Also, these cells possess the ability to differentiate into various lineages under specific culture conditions [46]. Stem cells are a great source of cells for tissue engineering, due to their capability for self-renewal and potential for multilineage differentiation. These features are important because most differentiated progenitor cells are difficult to obtain and possess limited capacities for proliferation [53].

In this study, hASCs seeded on electrospun PHB/PHB-HV fiber mesh were induced to differentiate into the endothelial lineage with the purpose of obtaining endothelial cells for enhancing the vascularization of bone grafts. The ability of hASCs to differentiate into endothelial cells was previously reported [54], [55]. We employed a specific medium to induce the differentiation and the MTT assay was performed to determine if the cells were viable and proliferative in the presence of the endothelial differentiation medium. MTT tests indicated that the cells cultured on electrospun PHB/PHB-HV fiber mesh proliferated in the presence of the endothelial medium, but the basal medium was significantly more effective in stimulating cell proliferation Research has demonstrated that the capacity to self-renew is unique to stem cells, and, as the differentiation stages increase, the proliferative potential diminishes [56]. Mature cells are unable to proliferate; however, the majority of lineages have a precursor or committed-progenitor stage in which proliferation and differentiation are balanced [57], [58]. The results indicated that the cells proliferate less in endothelial differential medium, and the proliferation was even less when the cells were cultured on the fiber mesh comparing to TCPS, suggesting that the electrospun PHB/PHB-HV was helping the compromise of the cells to the endothelial lineage. Although the cells in the differentiation medium proliferated less than those in the basal medium, the cells that were on the electrospun PHB/PHB-HV fiber mesh surface after 21 days were still viable, as demonstrated by the Calcein-AM assay.

In our differentiation system, the hASCs expressed endothelial markers, such as the vWF factor and VE-Cadherin. These markers are generally expressed when mesenchymal stem cells are induced to endothelial differentiation with medium containing VEGF and bFGF [54], [59]. Additionally, the expressions of those markers were enhanced on the cells cultured on electrospun PHB/PHB-HV fiber mesh. These expression are important for maintain the endothelial cells functions. VWF factor mediate the adhesion of blood platelets at sites of vascular injury and stabilizes clotting factor VIII. Also, vWF may help to anchor endothelial cells to ECM [60]. VE-Cadherin is a strictly endothelial specific adhesion molecule located at junctions between endothelial cells and plays an important role in the maintenance and control of endothelial cells contacts permitting the formation of a mature and stable vascular network [61].

Furthermore, the cells that were seeded onto the electrospun PHB/PHB-HV fiber mesh, when induced to endothelial differentiation, spread randomly and formed interconnected clusters with delineated of circular spaces among them. This capillary-like structure is similar to the structure formed when endothelial cells are cultivated in specialized conditions that mimics ECM structure, i.e collagen I gel supplemented with laminin [62], fibrin gel [63], [64], self-assembling peptide scaffold [65] and organotypic membrane systems [66]. These studies demonstrate that the capillary development depends on the interactions between endothelial cells and matrix proteins or biomaterials. In this sense, the structure of electrospun PHB/PHB-HV fiber mesh, that mimics the nanofiber structure of ECM can be favoring the endothelial differentiation.

Additionally, the differentiation of hASCs with VEGF also upregulates the expression of the VEGF receptor 2 (KDR), which plays a major rule in the *in vivo* angiogenesis and contributes, also, with matrix-metalloproteinase to the formation of capillary-like structures *in vitro* [59]. In resume, the differentiation results indicates that the structure of electrospun PHB/PHB-HV fiber mesh associated with specific growth factor, VEGF and bFGF, can improve the endothelial differentiation of hASCs. It has been reported the use of electrospinning technique as a good choice for developing structures for vascular tissue engineering. Many types of biomaterials, such as poly(l-lactide-co-e-caprolactone), polycaprolactone and silk, have been processed and have good properties that supports the attachment and proliferation of differently sources of endothelial cells [67]–[71]. However, these works use only endothelial cells and we demonstrated the possibility of stimulating the endothelial differentiation from hASCs. This enables the use of an autologous and proliferative source of cells.

In conclusion, this study demonstrated that the electrospun PHB/PHB-HV fiber mesh is a suitable and biocompatible material that can be employed in combination with endothelial differentiated cells. This new construct can be used for enhancing vascularization in engineered bone tissue. The differentiation of cells on the fiber mesh surface is interesting because the process can be conducted separately from other types of differentiation, which could prevent nonspecific differentiation. Furthermore, studies *in vivo* need to be performed to verify the efficiency of this new approach.

## Materials and Methods

### Preparation of polymers scaffolds

The PHB and PHB-HV polymers were obtained through the fermentation of sugar cane syrup from *Ralstonia eutropha* bacteria. The polymers were produced by PHB Industrial S.A. (PHBISA), Usina da Pedra, Serrana-SP, Brazil. The PHB/PHB-HV fiber mesh was produced in the 3B's Research Group - Portugal by the polymer processing technique named electrospinning. PHB and PHB-HV were dissolved in chloroform/dimethylformamide (30∶70) at a concentration of 5% (w/v). The resulting polymer solution was placed into a 5 ml syringe, which was fitted with a metallic needle having an internal diameter of 0.8 mm. The syringe was connected to a syringe pump (KDS100, KD Scientific) to control the flow rate. A positive electrode was connected to the needle. A flat aluminum sheet covering the ground plate was employed as the collector. A high voltage power supply was employed to generate the electrostatic field (0–25 kV). The capillary tip-to-collector distance and the flow rate were 15 cm and 1 ml/h, respectively. The applied voltage was 9 kV. The conditions for the electrospinning were optimized to obtain a continuous process and reproducible mesh morphology. The scaffolds were sterilized with γ irradiation from a Co^60^ source at 15 kGy, and the surface morphology was examined by scanning electron microscopy (Leica Cambridge S360 microscope). The SEM images of three different samples were analyzed using ImageJ software to determine the thickness and fiber diameter.

### Isolation of ASC from human adipose tissue

Human adipose tissue was obtained with written informed consent from healthy patients who had undergo liposuction surgery for an esthetic reasons in the “Plastic Surgery Center – Dr. Luiz Alberto Lamana” in Belo Horizonte, Minas Gerais, Brazil. The liposuction tissue was collected after obtaining informed consent from the patients according to procedures approved by Ethics Committee of Federal University of Minas Gerais. No diabetes, hepatitis, metabolic diseases, or other systemic complications were reported for these donors. The isolation and culture of human adipose tissue-derived stem cells (hASCs) was performed as described by Zuk [45]. Briefly, adipose tissues were washed with phosphate-buffered saline (PBS), and the extracellular matrix was digested with 0.15% type I collagenase for two hours and centrifuged at 1400 rpm for 10 min to obtain a pellet, which was incubated for two days at 37°C/5% CO_2_ atmosphere in DMEM (Dulbecco's Modified Eagle Medium) supplemented with 10% fetal bovine serum (FBS), 100 U/mL penicillin and 100 U/mL streptomycin (Basal medium). Following incubation, the tissue culture plates were washed to remove residual non-adherent cells and were maintained at 37°C/5% CO_2_ in the control medium and were cultured for 7 to 10 days until they reached confluence. The medium was changed every two days. The cells were then harvested by digestion with 0.05% trypsin-EDTA, centrifuged at 1400 rpm for 5 minutes, suspended in culture medium, and plated at a density of approximately 2×10^4^ cells/cm^2^. Cells were utilized at passage 4.

### Flow cytometry analysis

The cell surface antigens specific to hASCs were characterized by flow cytometry. Cells were harvested and washed with PBS. Approximately 5×10^5^ cells were incubated for 30 min at 4°C with the following primary antibodies: mouse anti-human CD29 (Santa Cruz Biotechnology), CD34 (Abcam), CD44 (Santa Cruz Biotechnology), CD45 (BD Bioscience), CD73 (PE-conjugated, BD Bioscience), HLA-ABC (FITC-conjugated, Abcam) and HLA-DR (FITC-conjugated, Abcam). After washing, the cells were incubated with a secondary antibody, the FITC-labeled anti-mouse IgG (Calbiochen), for 30 min at 4°C, washed again and suspended in PBS. As a control, cells were incubated with only the secondary antibody to exclude nonspecific binding. Quantitative analysis was performed using a FACScan argon laser cytometer (Becton Dickson, San Jose, CA). For each sample, 15.000 events were acquired and analyzed with the CELL QUEST software. Cell surface marker expression was determined by comparison with the isotype control on a histogram plot and data analysis was performed using WinMid 2.8 analysis software.

### Cell seeding

The hASCs at the passage 4 were seeded at a density of 1×10^5^ cells onto the electrospun PHB/PHB-HV fiber mesh with 1 cm^2^ and onto TCPS coverslip that had previously been placed in 24 well culture plates. The cells were allowed to attach to the substrates for 1 h, then 1 ml of fresh culture medium was added to each well. After, the cells were incubated in a humidified atmosphere at 37°C and 5% CO_2_.

### Morphological analysis using scanning electron microscopy (SEM)

In order to examine the morphology of hASCs, the cells were cultured for 7 days on the electrospun PHB/PHB-HV fiber mesh and the constructs prepared for SEM analysis. Basically, the samples were washed twice with PBS and fixed with 2.5% glutaraldehyde in 0.1 M sodium cacodylate buffer for 2 h, post-fixed in 1% osmium tetroxide for 2 h, dehydrated in increasing concentrations of ethanol (from 30%, 40%, 50%, 60%, 70%, 80% 90%, and 95% to 100%) and were critical point-dried. Next, the samples were mounted on aluminum stumps, coated with gold and examined with a scanning electron microscope at 15 KV (JEOL/JSM-6360LV from the Department of Metallurgy – UFMG).

### Cellular viability and proliferation

The viability and proliferation of the hASCs on electrospun PHB/PHB-HV fiber mesh was determined by the tetrazolium salt MTT (3-[4,5-dimethylthiazol-2-yl]-2, 5-diphenyltetrazolium bromide) assay, which is based on the reduction of tetrazolium salt to formazan crystals by the dehydrogenase present in living cells mitochondria [39]. At various culture times 7, 14 and 21 days after cell seeding, the medium was removed and 210 µl of fresh culture media and 170 µl of MTT solution (5 mg/mL in PBS) were added to each well, followed by incubation for 2 h at 37°C in a 5% CO_2_ atmosphere. The resulting formazan salts were solubilized with 210 µL of SDS-10% HCl (sodium dodecyl sulfate – hydrochloric acid) for 18 h at 37°C in a 5% CO_2_ atmosphere, and the optical density of the solution was evaluated with a microplate spectrophotometer at 595 nm. Cells cultured without the scaffold were employed as the control for the proliferation experiment, which evaluated the proliferation of the hASCs cultured with the basal medium and the endothelial differentiation medium.

### Morphology and distribution

For the assessment of the cell morphology and distribution and viability, hASCs were seeded onto electrospun PHB/PHB-HV fiber mesh at various time point over 7, 14 and 21 days and were incubated for 20 min in Tyrode's Hepes buffer (140 mmol/l NaCl, 0.34 mmol/l Na2HPO4, 2.9 mmol/l KCl, 10 mmol/l Hepes, 12 mmol/l NaHCO3, 5 mmol/l glucose, pH 7.4) containing 0.1 mM Calcein Acetoxymethylester (Calcein-AM). Calcein-AM is a non-fluorescent, cell-permeant compound that is hydrolyzed by intracellular esterases into the fluorescent anion calcein. The Calcein-AM stained scaffold was placed on a microscope slide and visualized through confocal microscopy (Zeiss LSM 510 Meta). The cellular viability was evaluated for cells cultured with the basal medium and the endothelial differentiation medium.

### Endothelial cell differentiation

The hASCs growing on electrospun PHB/PHB-HV fiber mesh scaffolds and on tissue culture polystyrene (TCPS) coverslips were induced to differentiate into the endothelial lineage by culturing with DMEM medium containing 2% FBS, 50 ng/mL VEGF and 10 ng/mL bFGF. The cells were cultivated for 7, 14 and 21 days in a humidified atmosphere at 37°C and 5% CO_2_. The medium was changed every two days.

### Immunofluorescence

After 21 days of endothelial differentiation, the cells were analyzed by immunofluorescence for the presence of endothelial cell markers. The cells were fixed with 4% paraformaldehyde for 15 min at room temperature (RT). Samples were then rinsed with 0.1% Triton for 10 min at RT. After washing with PBS, the samples were incubated overnight at 4°C with 1∶100 dilution of mouse anti-human vWF (VW92-3, Abcam) and 1∶20 dilution of FITC-labeled rabbit polyclonal anti-human VE-Cadherin (Abcam) primary antibodies. Following PBS washing, the samples that were incubated with the vWF antibody were next incubated with the anti-mouse Alexa Fluor 488 (Molecular Probes) secondary antibody for 1 h at RT. The nuclei were counterstained with 0.2 µg/mL Hoechst in PBS for 20 min. Next, the cells were washed with PBS, mounted and visualized through confocal microscopy (Zeiss LSM 510 Meta). The fluorescence intensity was calculated by measuring the intensity of the pixels using ImageJ software. The images were analyzed only in the green channel. Using ROI manager, a region was drawn around each cell to be measured, and other region with no fluorescence was drawn to be used for background subtraction. The mean of the intensity of the pixels of each cell was subtracted from the mean of the background and plotted on a graph, using GraphPad Prism 5.0. Each bar represents an average of 15 cells in three different regions.

### Reverse transcriptase-polymerase chain reaction (RT-PCR)

RT-PCR was performed to evaluate the relative mRNA levels expressed by the receptor of VEGF type 2 (VEGFR2) gene. Total cellular RNA was extracted from hASCs cultured on TCPS and PHB/PHB-HV fiber mesh up to 21 days (basal medium and differentiation medium) with Trizol (Invitrogen), as described by the manufacturer. Total RNA was treated with the RevertAid™ H Minus M-MuLV RT (Fermentas) to generate cDNA using an oligo(dT) adapter primer. Next, PCR amplification was performed for VEGFR2 and GAPDH cDNA. The primers used were VEGFR2 forward: 5′GGAATACCCCTTGAGTCC3′, VEGFR2 reverse: 5′CCTCCAACTGCCAATACC3′, GAPDH forward: 5′GGTATCGTGGAAGGACTCATGAC3′ and GAPDH reverse: 5′ATGCCAGTGAGCTTCCCGTTCAGC 3′. The PCR cycles were as follows: 94°C for 2 min, 94°C for 30 s, 56°C for 45 s and 72°C for 45 s (30 cycles), 72°C for 10 min. The RT-PCR products were analyzed through 1% agarose gel electrophoresis and visualized with ethidium bromide.

### Statistical analysis

All experiments were repeated 3 times in triplicate, and the data were presented as the mean ± SD. Statistical comparisons were performed using two-way analysis of variance (ANOVA) and Bonferroni's post-hoc test. P-values<0.05 were considered to be statistically significant.
